# Shared Molecular Signatures Across Neurodegenerative Diseases and Herpes Virus Infections Highlights Potential Mechanisms for Maladaptive Innate Immune Responses

**DOI:** 10.1038/s41598-019-45129-8

**Published:** 2019-06-19

**Authors:** Ana Caroline Costa Sa, Heather Madsen, James R. Brown

**Affiliations:** 1 Computational Biology, Human Genetics, Research and Development (R&D), GlaxoSmithKline (GSK), Collegeville, PA 19426 USA; 2 HIV Discovery, ViiV Healthcare, Research, Triangle Park, NC 27713 USA

**Keywords:** Neuroimmunology, Computational biology and bioinformatics

## Abstract

Growing evidence suggests that peripheral factors to the brain driving neuro-inflammation could affect Alzheimer’s Disease (AD) and Parkinson’s Disease (PD) severity. Herpes simplex virus type 1 (HSV1) infection has been associated with AD while other related viruses, including cytomegalovirus (CMV), Epstein-Bar virus and human herpesvirus 6 (HHV6), are known to infect neurons. Here we compare gene expression profiles between AD or PD patients to those afflicted with herpes viral infections as to discover novel potential neuro-inflammation pathways. We found multiple significant differentially expressed genes (DEGs) shared between AD/PD and viral infections including *SESN3* which has a genetic association for increased AD risk. Pathway enrichment analysis revealed viruses shared Oxidative Stress Defense System and LRRK2 pathways with AD and PD, respectively. We further processed our data to identify novel target and drug-repurposing opportunities including anti-inflammatory therapy, immune-modulators and cholinesterase inhibitors which could lead to new therapeutics paradigms for these neurodegenerative diseases.

## Introduction

Globally, Alzheimer’s disease (AD) and Parkinson’s disease (PD) are among the most common causes of severe and fatal dementia^1^. While these diseases’ pathological hallmarks are neuronal loss, extracellular senile plaques containing the peptide β amyloid, and neurofibrillary tangles for AD^1,2^, and the loss of neurons in the substantia nigra and elsewhere in association with the presence of Lewy bodies for PD^1,3^, recent data indicate that neuroinflammation is involved in the progression of both neurodegenerative disorders^2,3^. Neuroinflammation driven by activated microglial cells causes a vicious cycle of inflammatory reaction between microglia, astrocytes, and β-amyloid plaques leading to neuronal death^1^. Investigators have long suspected that pathogenic agents contribute to the onset and progression of AD and PD^4,5^. However, only recently it was demonstrated that increased concentrations of proinflammatory cytokines occur in the initial stages of neurodegenerative diseases^6^. Moreover, certain genetic variants in the chromosome 6 region that specifies immune response human leukocyte antigens (HLAs)^7^ are associated with PD.

Peripheral viral infections could elicit brain dysfunction through direct cytolytic effects on site or through whole body circulating inflammatory reactions^8^. Neurotropic viruses, such as arboviruses, influenza viruses and herpes viruses developed escape mechanisms from host immune surveillance enabling access to the central nervous system (CNS) which can result in long-lasting subclinical infections (reviewed in^9^). The systemic and local responses of the immune system to viral infection potentially contribute to neuronal damage, even in the absence of cell death^9^. Viruses can elicit CNS inflammation either by traversing a comprised Blood Brain Barrier (BBB), infecting peripheral nerves or broadly over-activating the peripheral innate and adaptive host immune system^10^.

Multiple studies report the association between Herpes simplex virus type 1 (HSV1) and Alzheimer’s disease^5,11,12^. Other members of the Herpesviridae family have been implicated, particularly EBV, CMV and HHV6^13–16^. Several viruses are capable of latent residency in the peripheral nervous system and target, in acute cases of encephalitis (for HSV1/2 and EBV), the same regions of the central nervous system (temporal and frontal cortex, and hippocampus) affected in AD^17^.

In this study, we hypothesize that comparisons of gene expression profiles from patients afflicted with either AD or PD with those profiles resulting from *Herpesviridae* infection, specifically CMV, EBV or HHV6, might reveal new associated neuro-inflammation pathways. We performed direct transcriptome profile comparisons between gene expression changes and enriched pathways in patients with *Herpesviridae* viral infection and AD/PD patients. To prioritize individual targets and pharmacological opportunities of intervention, we leveraged additional biological information such as human genetic disease associations and drug-repurposing analyses. We report here multiple human host genes and pathways that were significantly shared by human immune system responses to viral infections and neurodegenerative pathology.

## Results

### Data set selection

**Figure 1:**
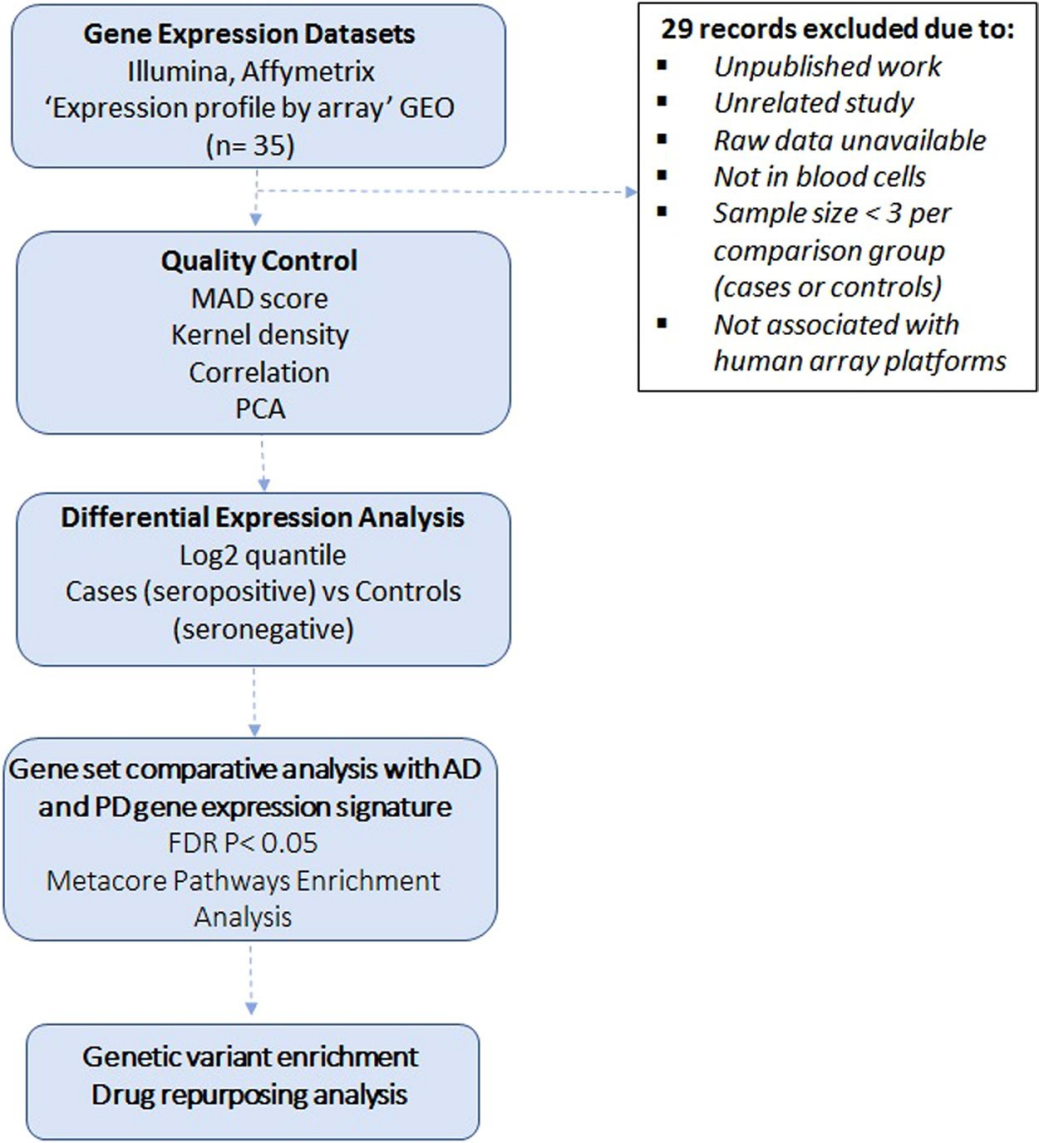
Flowchart of the cross-neurodegeneration (Alzheimer’s Disease and Parkinson’s Disease) and cross-viral infection (CMV, EBV and HHV6) transcriptome analysis pipeline. The computational analyses consist of six major steps which were presented in the blue boxes. Detailed criteria for each major step were described in the Methods.

As described in detail in Methods, our computational analyses involved six steps (Fig. 1): (1) NCBI Gene Expression Omnibus (GEO) database querying and selection of AD and PD and CMV, EBV and HHV6 infection datasets; (2) stringent quality control and normalization for each dataset (Table S1); (3) differential expression analysis of individual datasets for healthy controls versus diseased individuals; (4) comparisons of differential expressed genes (DEGs) and pathways enriched across AD, PD and viral infection profiles; (5) integration of genetic associations and tissue-specific gene expression data integration and (6) target repositioning hypotheses generation using EMBL-EBI ChEMBL and Connectivity Map (CMAP) databases.

We found 35 GEO datasets with gene expression profiles by microarrays for AD, PD and human host response to CMV, EBV and HHV6 (Table S1). We recalculated fold changes for AD and PD, and results are comparable with previous studies^18,19^. Based on the filtering criteria described in the Methods section, six datasets were selected (Table 1): GSE636063 (132 whole blood samples from healthy vs 135 AD patients^18^), GSE99039 (whole blood samples from 232 healthy vs 204 PD patients^19^), GSE81246 (peripheral blood mononuclear cells (PBMC) from 24 patients with latent CMV infection vs 10 patients with active disease^20^), GSE20200^21^ and GSE45829^22^ (7 B cell independent samples vs 7 EBV infected samples), and GSE40396 (22 whole blood samples from seronegative patients vs 10 patients seropositive for HHV6 with fever^23^). Table S1 summarizes all retrieved datasets along with the reasons for their inclusion or exclusion from our analyses.

**Table 1:** List of patient blood and *in vitro* B cells infected with EBV gene expression datasets selected in this study, and the number of samples and DEGs in each dataset.

Dataset	Phenotype	Cell type	PMID	Platform	Samples			DEGs
					Cases	Controls	Outliers	
GSE63063	Alzheimer’s Disease	Whole Blood	26343147	Illumina	134	132	6	906
GSE99039	Parkinson’s Disease	Whole Blood	28916538	Affymetrix	204	232	2	939
GSE81246	CMV host response	PBMCs	28031361	Affymetrix	10	24	0	1910
GSE20200	EBV host response	B cells	21147465	Exon Array	4	4	0	1491
GSE45829	EBV host response	B cells	23724103	Exon Array	3	3	0	8589
GSE40396	HHV6 host response	Whole Blood	23858444	Illumina	10	22	0	545

**Figure 2:**
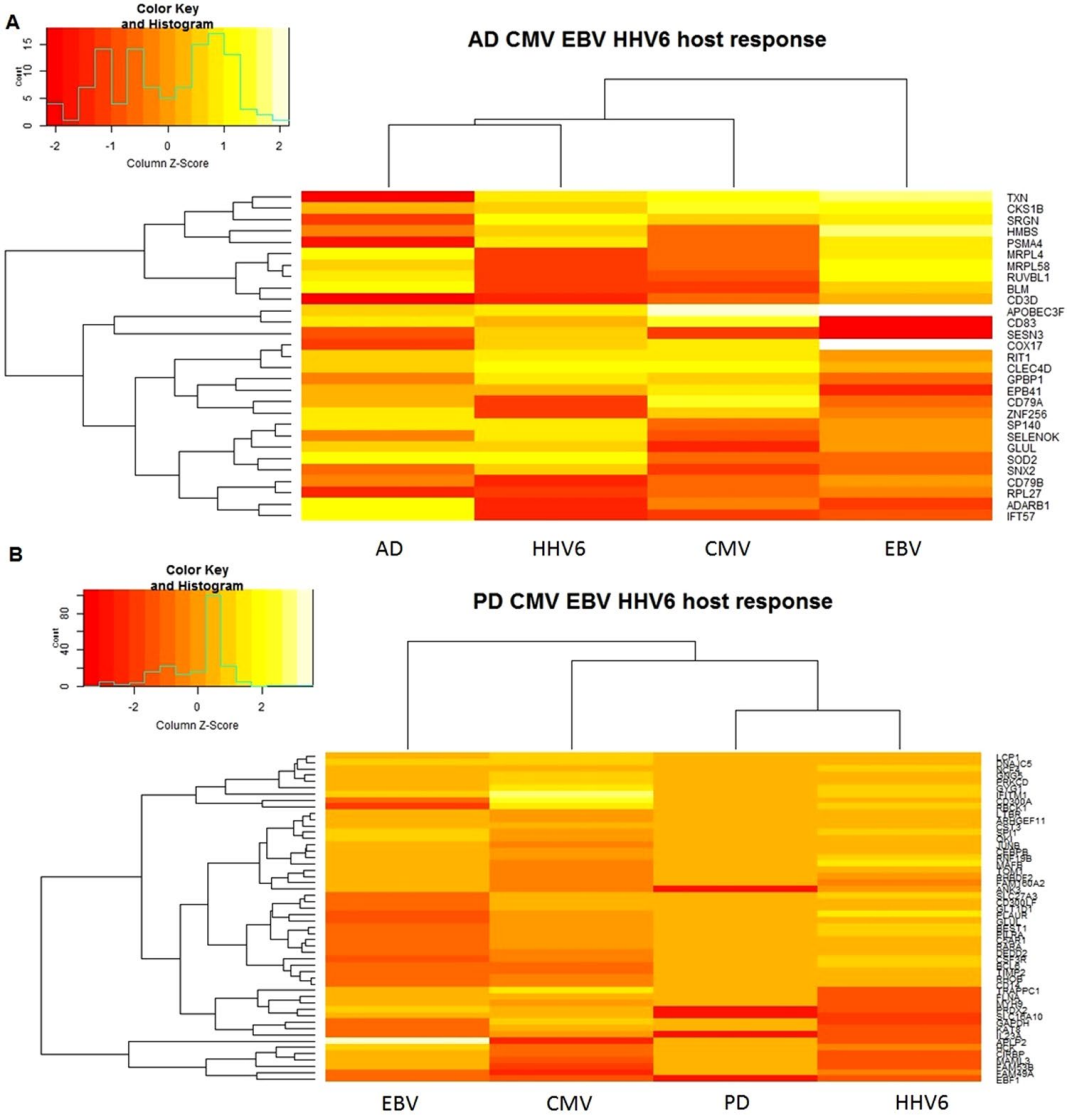
Heatmaps of subset of CMV, EBV and HHV6 DEGs shared with AD (**A**) and PD (**B**). For each DEG, log2 fold changes were indicated in the heatmap. The genes were clustered using the UPGMA method.

For each transcriptome dataset, we first determined the statistical significance of differentially expressed genes (DEGs) within each study by comparing disease to control samples. Subsequently, we compared individual study lists of significant DEGs to determine shared patterns in gene expression profiles for individual viruses compared to AD (Fig. 2A) and PD (Fig. 2B). This approach minimized any potential biases due to study differences in platforms or blood cell types.

### CMV infection and Alzheimer’s disease shared molecular markers

We identified 906 and 1,910 significant (False Discovery Rate adjusted [FDR-adj.] p-value < 0.05) DEGs in relation to AD and CMV human host response, respectively. Overall, 68 DEGs were shared in AD and CMV response signatures (Hypergeometric p-value (P_Hyper_) = 1.5 × 10^−7^; Table S2). *SESN3* was the most down-regulated gene in patients infected with CMV and in active disease status (CMV: Fold Change [FC] = −2.7, FDR-adj. p-value = 6.3 × 10^−11^; AD: FC = −1.3, FDR-adj. p-value = 5.0 × 10^−4^, Fig. 2A). Sestrin 3 controls intracellular response to reactive oxygen species^24^ and acts as a trans-acting genetic regulator of a pro-convulsant gene network in the human epileptic hippocampus^25^.

**Figure 3:**
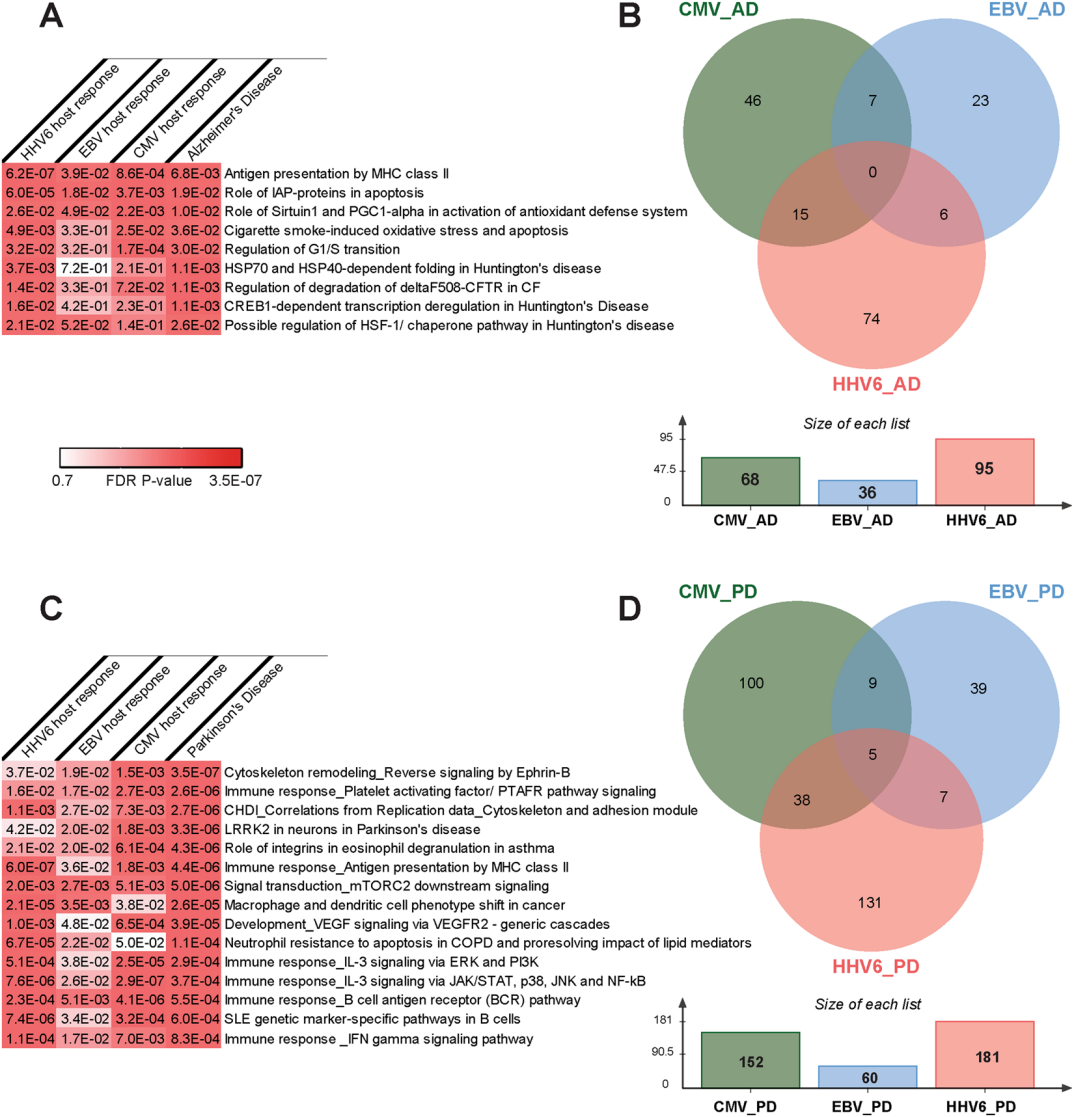
Statistically significant (adjusted FDR p-value ≤ 0.05) shared CMV, EBV and HHV6 human host response pathways and differentially expressed genes in AD (**A**,**B**) and PD (**C**,**D**), respectively.

**Figure 4:**
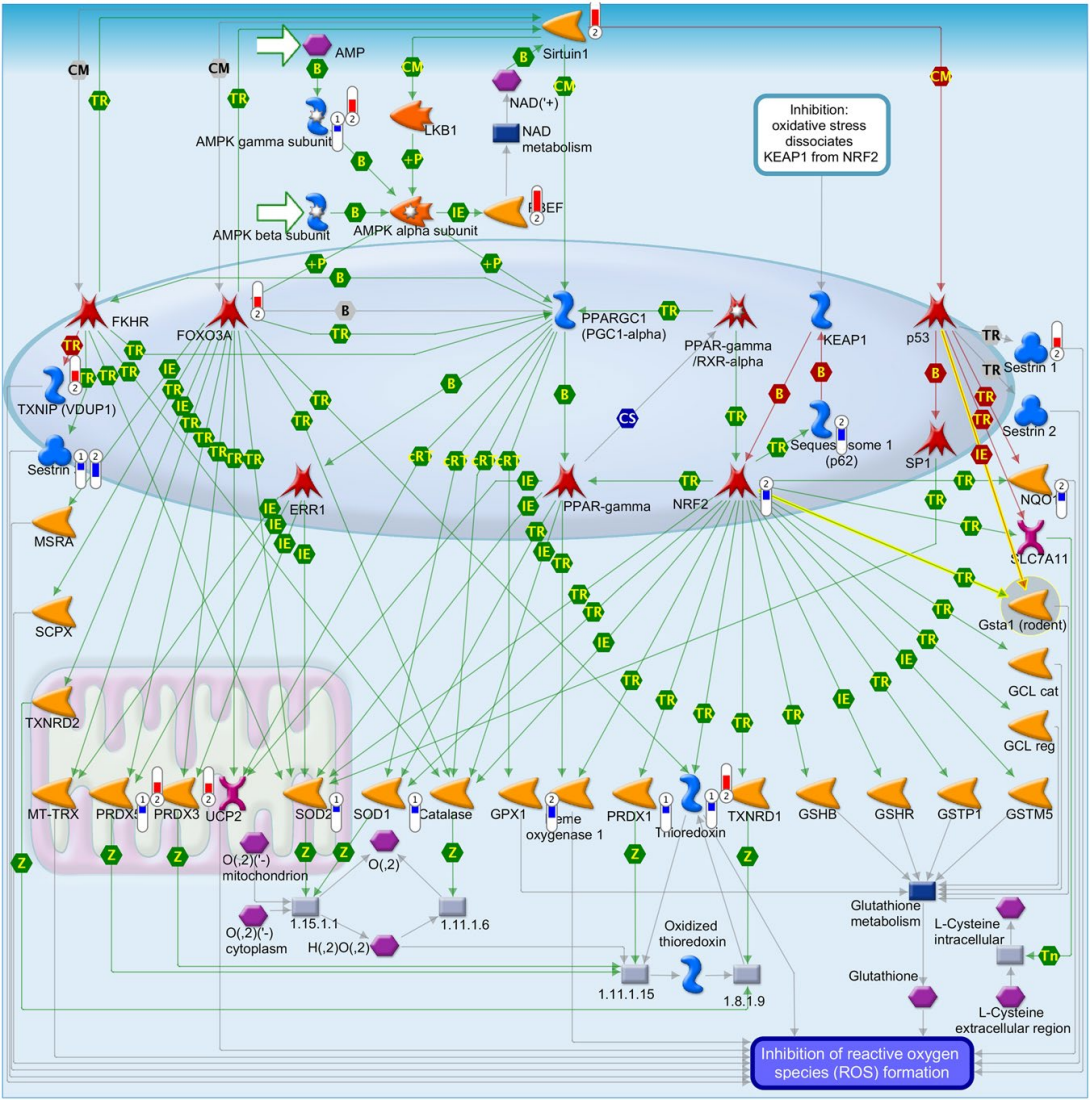
Pathway map for “Role of Sirtuin1 and PGC1-alpha in activation of antioxidant defense system”. Significant up-regulation of genes is denoted as up-pointing bars colored in red, and significant down-regulation of genes is denoted as down-pointing bars colored in blue. The height of the colored bar represents to the magnitude of the gene expression changes (fold change) between cases and controls.

The full list of significant DEGs associated with AD and CMV (906 and 1,910 DEGs respectively) were analyzed for enriched functional pathways using MetaCore/MetaBase (GeneGo) v6.0 (Thomson Reuters, https://portal.genego.com/). In total, 5 human canonical pathways were significantly enriched in both AD and CMV DEG lists (Fig. 3A). The most significant pathway was “Role of Sirtuin1 and PGC1-alpha in activation of antioxidant defense system” (Fig. 4) (AD: FDR-adj. p-value = 0.01; CMV: FDR-adj. p-value = 2.1 × 10^−3^).

### EBV infection and Alzheimer’s Disease shared molecular markers

We found 802 DEGs which were associated with EBV human host response. Of those, 36 genes were shared in AD and EBV host response signatures (P_Hyper_ = 2.0 × 10^−3^; Table S3). As in CMV host response comparison with AD transcriptional profiles, *SESN3* ranked as one of the top 20 genes associated with EBV human host response with same direction of association as seen in CMV human host response (EBV: FC = −6.9, FDR-adj. p-value = 1.1 × 10^−4^) (Fig. 2A).

Five canonical signaling pathways were enriched in both AD and EBV (Fig. 3A). Similar to that observed for CMV infection, the pathways “Role of Sirtuin1 and PGC1-alpha in activation of antioxidant defense system” (AD: FDR-adj. p-value = 0.01; EBV: FDR-adj. p-value = 0.049) and “Antigen presentation by MHC class II” were most significantly enriched in AD and EBV infection (AD: FDR-adj. p-value = 6.8 × 10^−3^; CMV: FDR-adj. p-value = 0.04).

### HHV6 infection and Alzheimer’s Disease shared molecular markers

We identified 1,698 genes associated with HHV6 human host response. Comparisons of transcriptional profiles yielded 95 genes shared in AD and HHV6 host response (P_Hyper_ = 0.038; Table S4). *IDO1* ranked as the top gene associated with HHV6 response (HHV6: FC = 4.5, FDR adj. p-value = 5.3 × 10^−5^; AD: FC = −1.08, FDR adj. p-value = 0.001, Fig. 2A). Indoleamine 2, 3-dioxygenase (IDO1) catalyzes the first and rate limiting step in the kynurenin pathway^26^, which has been implicated in neuroinflammation and neurodegeneration^27,28^.

For AD and HHV6 (906 and 1,698 DEGs respectively), there were 9 common significantly enriched canonical pathways of which “Antigen presentation by MHC class II” had the highest significance (AD: FDR-adj. p-value = 6.8 × 10^−3^; CMV: FDR-adj. p-value = 6.2 × 10^−6^) (Fig. 3A).

### Common genes and pathways across multiple viruses and Alzheimer’s Disease

We identified 28 genes that were associated with AD and host response to at least two of the three viruses investigated in this study (CMV, EBV or HHV6) (Fig. 3B). Thioredoxin (TXN) was the most down-regulated gene in patients with AD (FC = −1.44, FDR adj. p-value = 1.20 × 10^−3^), while up-regulated in patients with CMV (FC = 2.7, FDR adj. p-value = 3.0 × 10^−4^) and HHV6 active infection (FC = 1.51, FDR adj. p-value = 2.4 × 10^−3^) (Fig. 2A). Thioredoxin is crucial in maintaining a reduced oxygen intra-cellular environment and thus renders protection against oxidative stress^29^. For this reason, thioredoxin has been considered a promising early biomarker in the diagnosis of AD, suggesting the potential involvement of oxidative stress in the pathogenesis of the disease^30^.

Three pathways were significantly enriched in all host response viruses and AD datasets: “Antigen presentation by MHC class II”, “Role of IAP-proteins in apoptosis” and “Role of Sirtuin1 and PGC1-alpha in activation of antioxidant defense system” (Fig. 3A, Table S5). Two other pathways enriched in CMV and HHV6 host response as well as AD were “Induced oxidative stress and apoptosis in airway epithelial cells” and “Regulation of G1/S transition”.

### CMV infection and Parkinson’s Disease shared molecular markers

There were 939 genes associated with PD and 1910 genes associated with CMV host response. Of those, 152 DEGs were shared between PD and CMV host response (P_Hyper_ = 0.04; Table S6). Amyloid beta precursor like protein 2 (*APLP2)* was the most up-regulated gene in CMV host response (FC = 8.8, FDR adj. p-value = 4.1 × 10^−7^) and was also associated with PD (FC = 1.13, FDR adj. p-value = 3.6 × 10^−3^) (Fig. 2B). APLP2 belongs to the Alzheimer’s-associated amyloid beta-protein precursor gene family, which interacts with the synaptic release machinery, suggesting a role in neurotransmission^31^.

We found 28 canonical signaling pathways enriched in both PD and CMV using the full list of significant DEGs. The most significant pathways were “Integrin inside-out signaling in neutrophils” (PD: FDR-adj. p-value = 4.1 × 10^−11^; CMV: FDR-adj. p-value = 3.0 × 10^−3^) and “Inhibition of neutrophil migration by pro-resolving lipid mediators” (PD: FDR-adj. p-value = 4.1 × 10^−11^; CMV: FDR-adj. p-value = 3.4 × 10^−3^; Fig. 3C).

### EBV infection and Parkinson’s Disease shared molecular markers

Comparisons of transcriptional profiles yielded 60 genes shared in PD and EBV host response (P_Hyper_ = 0.02; Table S7). As seen in the CMV host response comparison with PD transcriptional profiles, *APLP2* ranked as one of the top 5 genes associated with EBV human host response (EBV: FC = −6.5, FDR-adj. p-value = 1.2 × 10^−4^; Fig. 2B).

From the list of genes associated with PD and EBV (939 and 802 DEGs respectively), there were 53 pathways significantly enriched in both diseases with “Reverse signaling by Ephrin-B” as the most significant one (PD: FDR-adj. p-value = 3.8 × 10^−7^; EBV: FDR-adj. p-value = 5.4 × 10^−3^; Fig. 3C).

### HHV6 infection and Parkinson’s Disease shared molecular markers

Overall, 181 DEGs were shared in PD and HHV-6 response signatures (P_Hyper_ = 6.4 × 10^−9^; Fig. 2B; Table S8). *IL1RN* was the most highly over-expressed gene in patients infected with HHV6 and significantly expressed in PD (HHV6: FC = 8.47, FDR-adj. p-value = 1.7 × 10^−6^; PD: FC = 0.27, p-value = 1.7 × 10^−3^). Interleukin 1 (IL-1) receptor antagonist (IL-1RN) is a naturally occurring anti-inflammatory agent that binds to the IL-1 receptor but lacks agonist activity and therefore functions like a competitive inhibitor of IL-1^32^.

Considering the total genes associated with PD and HHV6 (939 and 1697 DEGs respectively), 253 human canonical pathways significantly enriched in both DEG lists. As seen for CMV, the pathway entitled “Inhibition of neutrophil migration by pro-resolving lipid mediator” ranked as the most significant result (PD: FDR-adj. p-value = 4.3 × 10^−11^; EBV: FDR-adj. p-value = 3.0 × 10^−3^).

### Common genes and pathways across multiple viruses and Parkinson’s Disease

We identified 54 genes associated with PD and host response to at least two of the three viruses investigated in this study (CMV, EBV or HHV6) (Fig. 3D). *BCL6*, *GYG1*, *RBCK1*, *TIMP2* and *CIRBP* were common DEGs across all viruses tested and associated with PD. Tissue inhibitors of metalloproteinases (TIMPs) are endogenous inhibitors of matrix metalloproteinases (MMPs), and the aberrant expressions of MMPs are strongly associated with neuroinflammation and neuronal cell death^33^.

**Figure 5:**
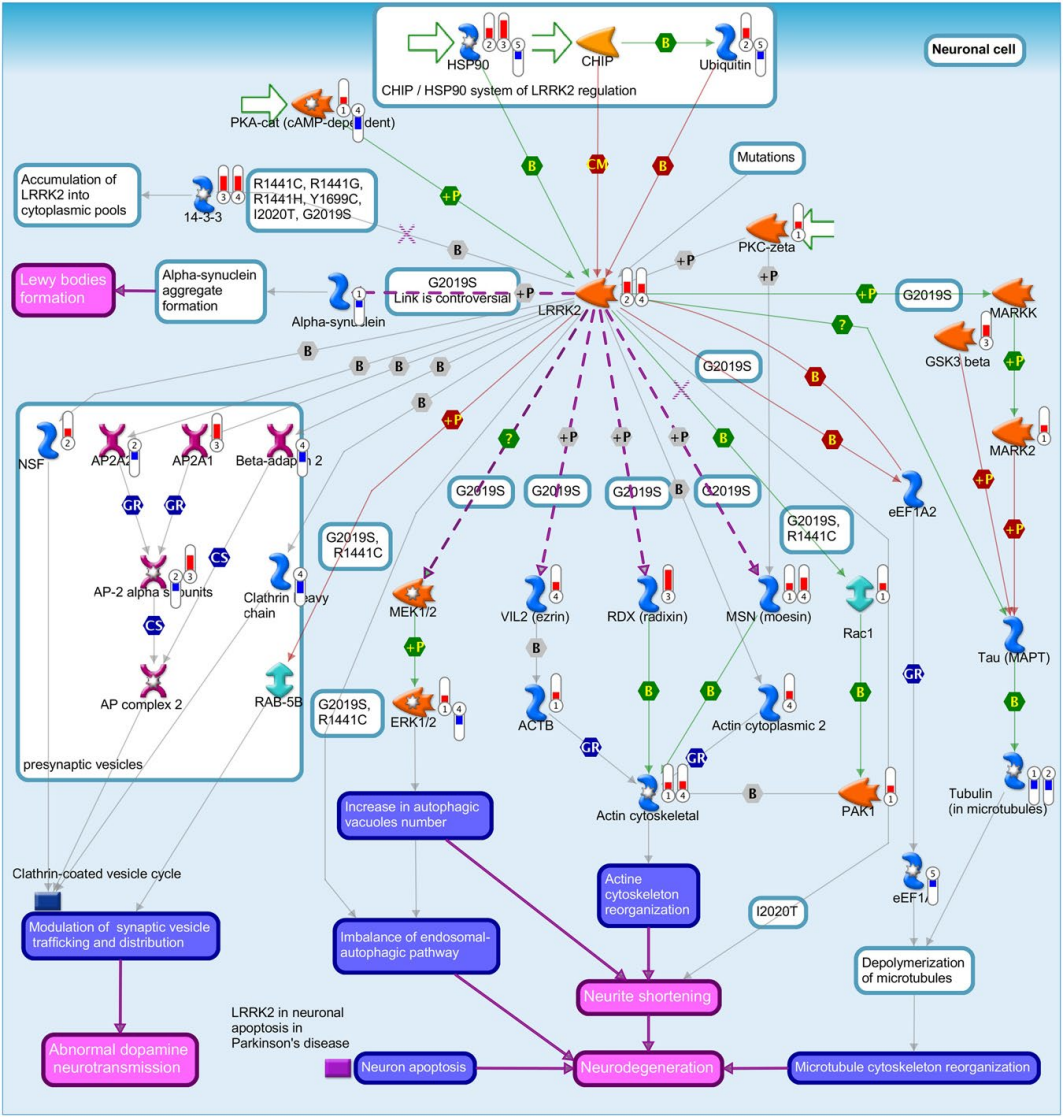
Pathway map for “LRRK2 in neurons in Parkinson’s disease”. Significant up-regulation of genes is denoted as up-pointing bars colored in red, and significant down-regulation of genes is denoted as down-pointing bars colored in blue. The height of the colored bar represents to the magnitude of the gene expression changes (fold change) between cases and controls.

In total, 15 human canonical pathways were significantly enriched from DEGs associated with PD and human host response to CMV, EBV and HHV6; many of which are involved in host immune response (Table S9). The most significant pathway was “Reverse signaling by Ephrin-B” (PD FDR-adj. p-value = 3.3 × 10^−6^; HHV6 FDR-adj. p-value = 3.7 × 10^−2^; EBV FDR-adj. *p-value* = 1.9 × 10^−2^; CMV FDR-adj. p-value = 1.5 × 10^−3^). Previous studies have indicated that ephrin signaling pathway is involved in the inflammatory process following CNS injury by serving roles in the maintenance of endothelial junction integrity and cytoskeletal structure. Remarkably, there was significant enrichment for several pathways commonly associated with PD and neurodegenerative diseases in general. These include the leucine rich repeat kinase 2 (LRRK2) pathway (PD FDR-adj. p-value = 3.7 × 10^−6^; HHV6 FDR-adj. p-value = 4.2 × 10^−2^; p-value = EBV FDR-adj. p-value = 2.0 × 10^−2^; CMV FDR-adj. p-value = 1.8 × 10^−3^; Fig. 5). The G2019S mutation within the LRRK2 kinase domain is the most common causal mutation in PD patients^34^, and it results in substantial increase in LRRK2 kinase activity^35^. The mechanism by which LRRK2-G2019S induces PD pathology remains unclear, although several studies have implicated this mutation in the dysregulation of autophagic function^36^.

### Control analyses for disease relevance of detected molecular signatures

To evaluate potential biases in our approach, we performed further analyses. First, we also compared CMV, EBV and HHV6 host response DEGs to genes associated with Huntington’s Disease (HD) and Type 2 diabetes mellitus (T2DM; FDR adj. p-value ≤ 0.05), as control analyses with another neurodegenerative condition (HD) and a disease unrelated to neurodegenerative and infectious disease (T2DM), to examine for potential spurious comparisons. We selected three publicly available datasets with peripheral blood gene expression samples: GSE9006^37^ (11 children diseased for T2DM and 23 healthy children), GSE69528^38^ (23 adults diseased for T2DM and 27 healthy controls) and GSE34721^39^ (150 samples from patients with HD and 70 samples from healthy controls). By contrast with the comparisons with AD and PD, these control analyses showed no statistically significant enrichment with T2DM or HD in the 3 datasets tested (P_Hyper_ > 0.1, Fig. S1) which suggests that our common molecular signatures between AD or PD and *Herpesviridae* infections are robust and non-spurious.

**Figure 6:**
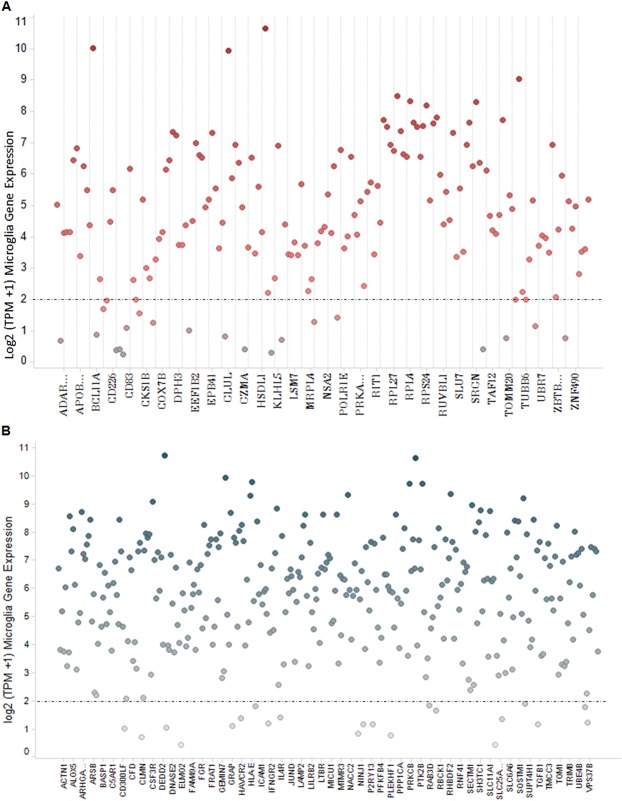
Microglia gene expression levels (log2 transcripts per million reads (TPM)) of CMV, EBV and HHV6 DEGs common with AD (**A**) or PD (**B**).

Second, since the gene expression datasets used in our analyses were obtained from blood samples of AD and PD patients we needed to evaluate the co-expression of shared viral host response genes with the most important immune function cell type found in the brain, the microglia. We re-analyzed and evaluated the gene expression profiles of 161 CMV, EBV or HHV6 host response genes shared with AD, and 329 genes shared with PD in 37 human microglia *post-mortem* samples, whose donors had history of normal cognitive function and no apparent neuropathological abnormalities^40^ (Fig. 6). We found the majority of genes (139 of 161 genes) 86.3% in AD and (308 of 329 genes) 93.6% in PD were actively expressed in human microglia (log2 TPM > 2) (Fig. 6). Of the DEGs shared by at least two of the three viruses, we found 82.1% (23 out of 28 genes) in AD and 90.7% (49 out of 54 genes) in PD were expressed in microglia. These findings lend further support for sampling of the blood as a surrogate for direct microglia gene expression profiling.

### Potential drug targets with human genetic evidence

It was previously reported that drug targets with robust human genetics support regarding disease pathology could boost success rates in clinical development^41^. Therefore, we surveyed the public genome-wide association studies present in the GWAS catalog for genetic evidence for the 172 and 329 DEGs that associated with host response to viruses (CMV, EBV or HHV6) and AD or PD, respectively. Each SNP was interposed to the linkage disequilibrium (LD) region upstream/downstream within 1 kb of the DEG coding region. A total of 19 genes were proximal to at least one SNP associated with neurodegenerative diseases (AD: 11 genes PD: 8 genes) (Table 2). Rs6430538, the most significant variant (p-value* = *8 × 10^−24^) associated with PD, is located near the gene *HNMT* on chromosome 2. HNMT encodes histamine N-methyltransferase which has a key leading role in histamine metabolism in the central nervous system^42^, and was pointed as a genomic biomarker for PD increased susceptibility^43^. Rs2373115, an intronic variant near GRB2 associated binding protein 2 (*GAB2)*, was the most significant variant (p-value = 1 × 10^−10^) associated with AD. Multiple genetic variants in the *GAB2* region are associated with late-AD onset, which could be involved in multiple pathways leading to the formation of neurofibrillary tangles^44^.

**Table 2:** List of 19 DEGs associated with viral host response and Alzheimer’s Disease or Parkinson’s Disease proximal to SNPs associated with neurodegenerative diseases in the GWAS catalog.

DEG	Gene Description	EBV DEG	CMV DEG	HHV6 DEG	Disease associated	Most significant variant	P-value	PUBMED IDs
HNMT	histamine N-methyltransferase		x		Parkinson’s disease	rs6430538	8.00E-24	28892059; 25064009; 22451204
SIPA1L2	signal induced proliferation associated 1 like 2			x	Parkinson’s disease	rs10797576	8.00E-13	28892059
RAB29	RAB29, member RAS oncogene family			x	Parkinson’s disease	rs947211	2.00E-12	19915576
ZNF626	zinc finger protein 626		x		Alzheimer’s disease	rs561655	7.00E-11	21460841
GAB2	GRB2 associated binding protein 2		x		Alzheimer’s disease	rs2373115	1.00E-10	17553421
VRK1	vaccinia related kinase 1			x	Alzheimer’s disease	rs150511909	4.00E-09	26830138
MRPL58	mitochondrial ribosomal protein		x	x	Alzheimer’s disease	rs9899728	2.00E-08	26913989
CLMN	calmin		x		Alzheimer’s disease	rs115102486	2.00E-08	26830138
PLEKHM1	pleckstrin homology and RUN domain containing M1	x			Parkinson’s disease	rs11012	6.00E-08	20070850
SESN3	sestrin 3		x	x	Alzheimer’s disease	rs3911569	3.00E-07	26830138
ADRM1	adhesion regulating molecule 1		x		Alzheimer’s disease	rs73310256	3.00E-07	26830138
MX2	MX dynamin like GTPase 2			x	Parkinson’s disease	rs78736162	3.00E-07	25663231
ACTN4	actinin alpha 4		x		Parkinson’s disease	rs62120679	6.00E-07	28892059
SQSTM1	sequestosome 1		x		Alzheimer’s disease	rs72807343	7.00E-07	24162737
RAB3D	RAB3D, member RAS oncogene family		x		Alzheimer’s disease	rs148273964	8.00E-07	26830138
RAB11FIP4	RAB11 family interacting protein 4	x			Alzheimer’s disease	rs142835438	8.00E-07	27770636
STAP1	Signal transducing adaptor family member 1		x		Parkinson’s disease	rs2242330	2.00E-06	17052657
IER2	immediate early response 2		x		Alzheimer’s disease	rs72998574	2.00E-06	27770636
GRN	granulin precursor			x	Parkinson’s disease	rs63750043; g.103432 C > T	NA	17923627

In addition, we identified several SNPs in close proximity (<1 KB to the coding region) of the 172 and 329 DEGs showed genetic association with other non-relevant conditions to neurodegenerative diseases. For example, 20 genes were proximal to SNPs associate with T2DM (Table S10).

### Drug repurposing analysis

The 401 targets identified in this study (172 and 329 DEGs associated with host response to viruses and AD and PD, respectively) were mapped to public compounds by searching the EMBL-EBI ChEMBL database for approved and marketed drugs targeting these genes. Overall, we identified 55 drug-target pairs in 20 DEGs (Table 3). Most genes were associated with multiple drugs, for example, TUBB6 was targeted by 12 unique compounds with diverse therapeutic indications such as oncology and acute coronary syndrome. Alfacalcidol, a vitamin D receptor (VDR) agonist with therapeutic indication for PD is also included in our list as a potential repurposing opportunity to treat peripheral drivers of neurodegeneration.

**Table 3:** List of launched drugs targeting DEGs associated with viral host response and Alzheimer’s Disease or Parkinson’s Disease.

Target	EBV_DEG	CMV_DEG	HHV6_DEG	Drug	Disease	Molecule type
VDR	x			ALFACALCIDOL	diabetic nephropathy	Small molecule
VDR	x			CHOLECALCIFEROL	type I diabetes mellitus; Parkinson’s Disease	Small molecule
VDR	x			CALCIPOTRIENE	psoriasis	Small molecule
VDR	x			ERGOCALCIFEROL	acute coronary syndrome	Small molecule
VDR	x			PARICALCITOL	chronic kidney disease	Small molecule
VDR	x			CALCITRIOL	Hypocalcemia	Small molecule
VDR	x			DOXERCALCIFEROL	secondary hyperparathyroidism	Small molecule
VDR	x			CALCIFEDIOL	secondary hyperparathyroidism	Small molecule
TYMP			x	TIPIRACIL	metastatic colorectal cancer	Small molecule
TUBB6		x		VINCRISTINE	acute lymphoblastic leukemia	Small molecule
TUBB6		x		DOCETAXEL	squamous cell carcinoma	Small molecule
TUBB6		x		PACLITAXEL	breast carcinoma	Small molecule
TUBB6		x		COLCHICINE	acute coronary syndrome	Small molecule
TUBB6		x		VINORELBINE	breast carcinoma	Small molecule
TUBB6		x		TRASTUZUMAB EMTANSINE	breast carcinoma	Antibody
TUBB6		x		BRENTUXIMAB VEDOTIN	lymphoma	Antibody
TUBB6		x		CABAZITAXEL	prostate carcinoma	Small molecule
TUBB6		x		VINBLASTINE	neoplasm	Small molecule
TUBB6		x		ERIBULIN	breast carcinoma	Small molecule
TUBB6		x		VINFLUNINE	neoplasm	Small molecule
TUBB6		x		IXABEPILONE	neoplasm	Small molecule
TOP1MT			x	TOPOTECAN	acute myeloid leukemia	Small molecule
RARA		x	x	TRETINOIN	acne	Small molecule
RARA		x	x	ADAPALENE	acne	Small molecule
RARA		x	x	ACITRETIN	psoriasis	Small molecule
RARA		x	x	ISOTRETINOIN	acne	Small molecule
RARA		x	x	ALITRETINOIN	Eczema	Small molecule
RARA		x	x	TAZAROTENE	psoriasis	Small molecule
RARA		x	x	ETRETINATE	psoriasis	Small molecule
PSMB10		x		BORTEZOMIB	multiple myeloma; Glycogen storage disease due to acid maltase deficiency	Small molecule
PSMB10		x		IXAZOMIB CITRATE	multiple myeloma	Small molecule
PSMB10		x		CARFILZOMIB	neoplasm	Protein
PDK3			x	SODIUM DICHLOROACETATE	lactic acidosis	Small molecule
IL4R		x		DUPILUMAB	Eczema	Antibody
IL23A		x	x	USTEKINUMAB	psoriasis	Antibody
IL17RA			x	BRODALUMAB	psoriasis	Antibody
IFNGR2			x	INTERFERON GAMA-1B	relapsing-remitting multiple sclerosis	Protein
IFNGR2			x	INTERFERON GAMMA-1B	idiopathic pulmonary fibrosis	Protein
HCK		x		BOSUTINIB	neoplasm	Small molecule
FGR	x		x	DASATINIB	chronic myelogenous leukemia	Small molecule
EPHB6	x		x	VANDETANIB	thyroid carcinoma	Small molecule
EPHB6	x		x	PREDNIMUSTINE	lymphoma	Small molecule
CSF3R		x	x	PEGFILGRASTIM	breast carcinoma	Protein
CSF3R		x	x	FILGRASTIM	myocardial infarction	Protein
CSF3R		x	x	LIPEGFILGRASTIM	lymphoma	Small molecule
CD52				ALEMTUZUMAB	diabetes mellitus	Antibody
CD3D		x	x	BLINATUMOMAB	acute lymphoblastic leukemia	Antibody
CD3D		x	x	MUROMONAB-CD3	immune system disease	Antibody
CD3D		x	x	CATUMAXOMAB	neoplasm	Antibody
BCR			x	PONATINIB	neoplasm	Small molecule
ALOX5			x	SULFASALAZINE	rheumatoid arthritis	Small molecule
ALOX5			x	ZILEUTON	asthma	Small molecule
ALOX5			x	MESALAMINE	ulcerative colitis	Small molecule
ALOX5			x	BALSALAZIDE	ulcerative colitis	Small molecule
ALOX5			x	OLSALAZINE	ankylosing spondylitis	Small molecule

CMAP is another drug repurposing approach which deploys the anti-correlation relationship across disease gene expression signatures and pharmacological *in vitro* perturbations^45^. We performed separate analyses using CMV, EBV and HHV6 human host response gene expression signatures to assess anti-correlation of approximately 5000 small-molecule compounds and 300 reagents from the Broad Institute public library (www.broadinstitute.org/connectivity-map-cmap). Overall, 16, 24 and 16 compounds were significantly anti-correlated to the CMV, EBV and HHV6 host response signature respectively (p-value < 0.05, Specificity < 0.1; Table 4). Of those, 14 compounds (highlighted in Table 4) showed evidence in the literature of neuro-protection to Alzheimer’s or Parkinson’s Disease through multiple mechanisms, such as dopamine receptor agonism, monoamine oxidase and cholinesterase inhibition. In addition, multiple compounds identified showed anti-inflammatory properties, which were previously considered potential pharmacological options for AD prevention^46^.

**Table 4:** List of the Broad Institute public library of compounds associated with gene targets in CMV, EBV or HHV6 human host response based on CMAP^45^ analysis of contrary gene expression profiles.

Compound	Mechanism of Action	Indication	CMV Host response signature			EBV Host response signature			HHV6 Host response signature		
			Compound Score	Enrichment Score	P-Value	Compound Score	Enrichment Score	P-Value	Compound Score	Enrichment Score	P-Value
Quinostatin	PI3-Kinase/mTOR inhibitors	Oncology	1.00	−0.87	0.0337	1	−0.94	0.0085	NA	NA	NA
Cortisone	Corticosteroid Hormone Receptor Agonists	Anti-inflammatory	0.50	−0.88	0.0285	NA	NA	NA	NA	NA	NA
Quinethazone	Sodium/chloride tranporter inhibitor	Antihypertension	0.50	−0.84	0.0492	NA	NA	NA	NA	NA	NA
**Metrifonate**	**Cholinesterase inhibitor**	**Neuro protection** ^86, 87^	**0**.**37**	−**0**.**95**	**0**.**0054**	**NA**	**NA**	**NA**	**NA**	**NA**	**NA**
Cicloheximide	Protein synthesis inhibitor	Antibiotics	0.33	−0.99	0.0002	0.33	−0.93	0.0107	NA	NA	NA
**Anisomycin**	**MAP kinase activator**	**Neuro protection** ^71^	**0**.**33**	−**0**.**97**	**0**.**0023**	**0**.**33**	−**0**.**96**	**0**.**0038**	**NA**	**NA**	**NA**
**Molindone**	**Dopamine receptor antagonist**	**Neuro protection** ^69^	**0**.**33**	−**0**.**94**	**0**.**0059**	**NA**	**NA**	**NA**	**0**.**33**	−**0**.**90**	**0**.**0216**
Hydroflumethiazide	Na-Cl cotransporter inhibitor	Antihypertensive	0.33	−0.94	0.0077	NA	NA	NA	NA	NA	NA
**Pronetalol**	**Adrenoreceptor blocker (beta)**	**Neuro protection69**	**0**.**33**	−**0**.**93**	**0**.**0089**	**NA**	**NA**	**NA**	**NA**	**NA**	**NA**
Picotamide	Eicosenoid receptor antagonist	Anti-inflammatory	0.33	−0.93	0.0103	NA	NA	NA	NA	NA	NA
Mephenytoin	Sodium channel blocker	Antihypertensive	0.33	−0.89	0.0231	NA	NA	NA	NA	NA	NA
**Dipivefrine**	**Adrenergic agonist**	**Neuro protection** ^69^	**0**.**33**	−**0**.**87**	**0**.**0343**	**NA**	**NA**	**NA**	**NA**	**NA**	**NA**
Etamsylate	Prostaglandin synthesis inhibitor	Anti-inflammatory	0.33	−0.85	0.0422	NA	NA	NA	NA	NA	NA
**Mebeverine**	**Phosphodiesterase inhibitor**	**Neuro protection** ^88^	**0**.**33**	−**0**.**85**	**0**.**045**	**NA**	**NA**	**NA**	**NA**	**NA**	**NA**
**Prasterone**	**Estrogen receptor (ER) agonists Androgen receptor (AR) agonists**	**Neuro protection** ^89^	**0**.**33**	−**0**.**85**	**0**.**0467**	**NA**	**NA**	**NA**	**NA**	**NA**	**NA**
**Pirenzepine**	**Muscarinic M1 receptor antagonist**	**Neuro protection** ^69^	**0**.**30**	−**0**.**95**	**0**.**0045**	**NA**	**NA**	**NA**	**NA**	**NA**	**NA**
Calmidazolium	Calmodulin binding inhibitor	Immunosuppressant	NA	NA	NA	1.00	−0.9023	0.0193	NA	NA	NA
Antazoline	Histamine H1 receptor antagonist	Allergy	NA	NA	NA	0.33	−0.8696	0.0342	NA	NA	NA
Beta-escin	Vasoconstriction	Cardiovascular Agent [Pubchem]	NA	NA	NA	0.33	−0.7912	0.0181	NA	NA	NA
Betahistine	Histamine H1 receptor agonist	Anti-vertigo	NA	NA	NA	0.33	−0.8763	0.0312	NA	NA	NA
Cephaeline	Anti-neoplastic	Oncology	NA	NA	NA	0.33	−0.9537	0.0002	NA	NA	NA
Cetirizine	Histamine H1 receptor antagonist	Allergy	NA	NA	NA	0.33	−0.8937	0.023	NA	NA	NA
Dequalinium chloride	Anti-bacterial	Antibiotics	NA	NA	NA	0.33	−0.9666	0.0021	NA	NA	NA
Domperidone	Dopamine receptor D2 antagonist	Antiemetic	NA	NA	NA	0.33	−0.8436	0.0495	NA	NA	NA
Emetine	anti-parasitic	Anti-parasitic	NA	NA	NA	0.33	−0.9978	0	NA	NA	NA
Felodipine	Calcium channel (L-type) blocker	Antihypertensive	NA	NA	NA	0.33	−0.6897	0.0066	NA	NA	NA
Flunarizine	Sodium channel antagonist; Calmodulin binding and H1 antagonist	Severe Migraine	NA	NA	NA	0.33	−0.9181	0.0137	NA	NA	NA
**Metergoline**	**Serotonin and Dopamine receptors ligand**	**Pychoactive (Drug Bank)**	**NA**	**NA**	**NA**	**0**.**33**	−**0**.**9576**	**0**.**0036**	**NA**	**NA**	**NA**
Natamycin	Anti-infective agent	Anti-parasitic	NA	NA	NA	0.33	−0.9229	0.0121	NA	NA	NA
**Pargyline**	**Monoamine oxidase inhibitor**	**Neuro protection** ^87^	**NA**	**NA**	**NA**	**0**.**33**	−**0**.**937**	**0**.**0081**	**NA**	**NA**	**NA**
Perhexiline	CPT inhibitor	Cardiovascular Agent [Pubchem]	NA	NA	NA	0.33	−0.8921	0.0238	NA	NA	NA
Phenyl propanolamine	Adrenoreceptor agonist (alpha)	Allergy	NA	NA	NA	0.33	−0.9258	0.0113	NA	NA	NA
**Piribedil**	**Dopamine receptor agonist**	**Parkinson’s Treatment**	**NA**	**NA**	**NA**	**0**.**33**	−**0**.**9197**	**0**.**0132**	**NA**	**NA**	**NA**
Pyrvinium	Anthelmintic	Anti-parasitic	NA	NA	NA	0.33	−0.7906	0.0039	NA	NA	NA
Saquinavir	HIV Protease inhibitor	Anti-retroviral	NA	NA	NA	0.33	−0.8712	0.0335	NA	NA	NA
Talampicillin	Peptidoglycan synthesis inhibitor	Antibiotics	NA	NA	NA	0.33	−0.8465	0.0476	NA	NA	NA
Tretinoin	Retinoid	Skin related conditions	NA	NA	NA	0.33	−0.4968	0.0017	NA	NA	NA
Cyclopenthiazide	Sodium Chloride Symporter Inhibitor	Antihypertensive	NA	NA	NA	NA	NA	NA	0.33	−0.9846	0.0004
Alcuronium chloride	Cholinergic receptor antagonist	Muscle relaxant	NA	NA	NA	NA	NA	NA	1.00	−0.956	0.0036
**Pergolide**	**Dopamine receptor agonist**	**Pychoactive (Drug Bank)**	**NA**	**NA**	**NA**	**NA**	**NA**	**NA**	**0**.**33**	−**0**.**957**	**0**.**0036**
Staurosporine	Protein kinase inhibitor	Oncology	NA	NA	NA	NA	NA	NA	1.00	−0.8538	0.006
**Hemicholinium**	**Acetylcholine stores depletor**	**Neuro protection** ^1^	**NA**	**NA**	**NA**	**NA**	**NA**	**NA**	**0**.**33**	−**0**.**9296**	**0**.**0094**
Suramin sodium	Topoisomerase inhibitor	Oncology	NA	NA	NA	NA	NA	NA	0.45	−0.9171	0.013
Pyrantel	antihelmintic	Anti-parasitic	NA	NA	NA	NA	NA	NA	0.33	−0.9011	0.0191
Arachidonyl trifluoromethane	Phospholipase A2 inhibitor	Anti-inflammatory	NA	NA	NA	NA	NA	NA	1.00	−0.9004	0.0193
Triprolidine	Histamine H1 receptor antagonist	Allergy	NA	NA	NA	NA	NA	NA	0.33	−0.8889	0.0242
Ethambutol	Chelating agent	Antibiotics	NA	NA	NA	NA	NA	NA	0.30	−0.886	0.0255
**Minaprine**	**5-HT2 receptor inhibitor; Dopamine receptor agonist**	**Parkinson’s Treatment**	**NA**	**NA**	**NA**	**NA**	**NA**	**NA**	**0**.**37**	−**0**.**8763**	**0**.**0303**
Nimesulide	Cyclooxygenase-2 inhibitor	Anti-inflammatory	NA	NA	NA	NA	NA	NA	0.33	−0.8728	0.0322
Ganciclovir	DNA synthesis inhibitor	Antibiotics	NA	NA	NA	NA	NA	NA	0.33	−0.8612	0.0382
Sparteine	Anti-inflammatory diuretic Anti-infective agent	Anti-inflammatory	NA	NA	NA	NA	NA	NA	0.33	−0.85	0.0446
Pentoxifylline	Phosphodiesterase inhibitor	Intermittent Claudication	NA	NA	NA	NA	NA	NA	0.33	−0.849	0.0454

Compounds with neuro protection evidence were highlighted (in bold).

## Discussion

Understanding the causal basis for neurodegenerative diseases is challenged by its extended preclinical stage, and the unfeasible task to sample brain tissues routinely. Currently, it is known that neurodegenerative diseases, such as AD and PD, could result from multiple risk factors including genetic susceptibility^47,48^, age^49^, and toxins and inflammatory responses as environmental triggers for microglia and astrocyte activation^50^. In this context, pervasive viral infections could precipitate peripheral inflammatory reactions or immune dysregulation that are often associated with AD and PD^9,36,51^. We report here a systematic study of common molecular markers between viral perturbations to human immune response and clinical AD and PD. Our strategy was to examine multiple public transcriptome datasets from patients seropositive/seronegative for CMV, EBV or HHV6, and AD/PD patients with the goal of identifying novel biology mechanisms suited for therapeutic modulation.

The concept of utilizing datasets with blood samples for the detection of disease associated molecular changes in gene expression relies on the natural role of peripheral blood cells in immune response to circulating pathogens. This enabled our blood-to-blood sample gene expression comparisons between human host response to CMV, EBV or HHV-6 infection to that of AD and PD patients. In addition, recent studies demonstrated significant correlation in gene expression between multiple brain tissues and peripheral blood cells^52–56^. We confirmed that the majority of DEGs from the blood are also actively expressed in human microglia. Therefore, we feel there is validity in our approach of inferring genes and pathways involved in AD/PD pathology through comparative blood differential gene expression analyses with host response to viral pathogens.

Our results provide evidence of the involvement of oxidative stress mechanisms in the pathologies of our representative viruses and AD through the activation of the Sirtuin and PGC1-alpha pathway. In addition, *SESN3* and *TXN*, which play important roles in this pathway, ranked among the top genes associated with CMV and EBV, and CMV and HHV6 host responses, respectively. Further support is provided by genetic evidence from GWAS which show an association of the SNP rs3911569 located near the gene *SESN3* with a 5-fold increased risk for AD. These findings support the emerging “mitochondrial cascade hypothesis” based on growing evidence for AD-related mitochondrial dysfunction^57^, and the potential impact of CMV, EBV and HHV6 host response in oxidative stress.

Our analyses also highlighted *BCL6*, *GYG1*, *RBCK1*, *TIMP2* and *CIRBP*, which were DEGs shared between all viruses and PD. TIMP2 was associated with neuroprotection through inhibition of matrix metalloproteinases^33^, which were involved in neuropathological processes such as inflammation, BBB damage and neuronal cell death, leading to multiple CNS disorders such as PD^58^. To our knowledge, none of the other genes have been previously linked to PD, neurodegeneration or neuroinflammation. BCL6, a sequence specific transcriptional repressor which is a key player in B cell differentiation, has recently gained attention due to the association of EBV latent proteins with *BCL6* down-regulation^59^. These findings have implications for emerging strategies targeting B cell differentiation but how they could influence neurodegeneration still needs further investigation.

Recent studies show that LRRK2, a kinase mutated in PD clinical cases^60–62^, modulates inflammation in response to different pathological stimuli. LRRK2 plays a potential role in cytoskeleton remodeling and vesicle trafficking in microglia cells toward a pro-inflammatory state and, consequently, neurodegeneration^63^. The LRRK2 pathway was significantly enriched from DEGs associated with PD and human host response to CMV, EBV and HHV6. LRRK2 gene expression is regulated by IFN-γ and potentially mediates immune responses to pathogens^64,65^. Recently, we reported that this pathway was also linked to human host response to *Mycobacterium tuberculosis*^66^. LRRK2 knock-outs in mouse models, displays phenotypes of hyperactive immune responses and increased risk to inflammatory bowel disease by regulating the transcriptional regulatory protein nuclear factor of activated T cells^67^. Our findings further support the potential roles of LRRK2 in host response to infection and neurodegeneration.

By mapping the 401 DEGs identified in this study to compounds listed in the ChEMBL database, we identified 55 drug-target pairs for 20 genes. Of those, 12 drug-target pairs showed primary therapeutic indication for auto-immune disease or chronic inflammatory conditions, such as psoriasis and rheumatoid arthritis. These results highlight the role of immune dysregulation in neurodegeneration, particularly, in AD and PD^68^. Thus, our findings suggest the use of immunomodulators as potential therapeutic strategies for AD and PD. Pro-inflammatory cytokines and chemokines as well as reactive oxygen and nitrogen species secreted by activated microglia can trigger a neurotoxic cascade leading to neuronal lesions and significant damage to the CNS. Therefore, therapies targeting neuroinflammation either directly or indirectly warrant further investigation^68^.

From our CMAP analysis, we identified several clinically used drugs that could be potentially repurposed for targeting human host factors in CMV, EBV and HHV6 infections. Overall, 14 of those compounds showed evidence in the literature of neuro-protection to AD or PD through multiple mechanisms, such as dopamine receptor agonism, and monoamine oxidase and cholinesterase inhibition^69–71^. Moreover, other CMAP compounds identified showed anti-inflammatory properties, many of which have shown promising results in experimental models of the disease^70,72^. These findings suggest several relevant mechanisms pertinent to both viral infection and neurodegeneration that need to be further explored.

Multiple epidemiological reports have associated AD or PD with diverse bacterial and viral pathogens^4,5,73^. Most of them connect *Herpesviridae* to AD, particularly HSV-1^74–76^, EBV, CMV, and HHV6^13–16^. In aggregate, these studies are suggestive of a viral contribution to neurodegenerative diseases although their findings offer little insight into potential mechanisms. Recently, Readhead *et al*.^77^ compared computational networks between AD and the RNA-Seq abundance of multiple viruses. Their findings implicate HHV6 and HHV7 contribution to the development of neuropathology and AD. Differently, our study provides a direct gene expression comparison of changes in expression in patients with documented evidence for viral infection (and active disease) and AD/PD. Arguably, using host gene expression signatures might be a more “agonistic” approach which overcomes the limitations of “virus hunting” for specific pathogens and could reveal the participation of both known and unknown viruses (or other pathogens) in neurodegenerative disease pathology based on overall host response.

Our study has some limitations to be considered. Most importantly, we were limited by the sample size and quality of publicly available datasets. For instance, we wished to investigate HSV-1 human host response comparing blood gene expression with the AD/PD DEGs/pathways but none of the available datasets were generated from patients. Moreover, the results presented here are not enough to conclusively prove causality relationship between viral host response and neurodegeneration. For that to occur, further clinical trials and interventional studies are necessary. Lastly, drug repurposing compounds obtained from CMAP analyses are derived from Broad Institute gene expression data on fibroblasts and tumor cell lines, which may not be the most relevant tissue for this study. Validating these results on microglia or brain tissues or even specific blood cells would be ideal.

Our study adds to the growing evidence of the role of immune dysfunction in neurodegenerative diseases. Moreover, gene expression systematic comparisons between host response to EBV, CMV and HHV6 and AD/PD provide new insights into host genes and pathways important for neurodegeneration and convey potential drug repurposing opportunities promoting neuroprotection. Experimental validation of the pharmacologic interventions proposed here would constitute the next stage in the drug development for the proposed targets and compounds. Further evolution of this paradigm shift viewing peripheral immunity dysregulation as a potential driver of neurodegeneration could lead to novel therapeutic approaches for the treatment of PD and AD.

## Methods

### Selection of gene expression datasets

Data analysis workflows broadly followed our previously published studies on human host response to various intra-cellular residing pathogens including bacteria^78^, viruses^79^ and tuberculosis^66^. Gene Expression Omnibus (GEO) database (as of June 2018) was queried for human blood microarray gene expression datasets in response to *Herpesviridae* infection, Alzheimer’s and Parkinson’s Disease. The specific search terms used were: “HSV”, “EBV”, “HHV6”, “CMV”, “Alzheimer’s Disease”, “Parkinson’s Disease”, “Homo sapiens”, and “Whole blood”. The retrieved datasets were filtered based on the following criteria: gene expression profiles from published studies which were: 1) raw data available and derived from human cells of AD/PD or single virus infected patients; 2) there was at least one control group (healthy subjects) and one diseased group and; 3) data originated from human array platforms. Additionally, type 2 diabetes mellitus (T2DM) and Huntington’s Disease (HD) datasets were included to allow gene expression comparisons with unrelated neurodegenerative and non-infectious diseases and serve as controls for spurious comparisons. Table S1 summarizes all retrieved datasets along with the reasons for their inclusion or exclusion from our analyses.

Raw (intra-slide normalized) gene expression data, study design table and annotation table of each dataset were obtained from the GEO/ArrayExpress databases and processed using ArrayStudio v10.0 (OmicSoft, USA). The datasets retrieved are microarray datasets obtained from the following platforms: Illumina Human HT, Affymetrics Human Genome U133 and Affymetrics Exon Array (Table S1). Several datasets (GSE42834, GSE56153, GSE31348 and GSE36238) were further excluded due to both a noisy kernel density plot and low within group pairwise correlation (correlation cutoff 0.9, Table S1), but were included as independent datasets for validation purposes. After quality filtering, six microarray datasets (GSE636063, GSE99039, GSE81246, GSE202007, GSE458298, and GSE40396) from either whole blood or peripheral blood mononuclear cells (PBMCs) were retained for further analysis.

### Data processing and statistical analysis

Quality Control analyses were performed in all datasets selected^66^. Data imported was previously normalized. Intra-slide normalization was assessed by: 1) kernel density; 2) Principal Component Analysis or PCA (showing divergent samples within groups); 3) Median Absolute Deviation (MAD) score and; 4) within group pairwise correlation. Samples were considered outliers if failed at least two of these assessments. Samples irrelevant to our study design (such as samples from host response to bacteria and viruses other than HHV6 in GSE40396) were also excluded. In total, 6 samples from AD, 2 samples from PD datasets were excluded. All samples in CMV, EBV, HHV6 and T2DM datasets passed QC.

For each dataset, scale quantile inter-slide normalization (fixed target median value to 500), log2 transformation and probe differential expression analysis was performed in ArrayStudio v10.0 (OmicSoft, USA). When more than one probe mapped to a gene, the expression value of the lowest p-value was used for that gene (the “aggregate” R function was applied). Differentially expressed genes (DEG) passed the false discovery rate adjusted [FDR-adj.] p-value threshold of 0.05. The AD or PD DEG list was compared with the list of DEGs associated with CMV, EBV or HHV6 host response to identify shared gene expression signatures. The statistical significance of the overlap between AD/PD DEGs with CMV, EBV or HHV6 DEGs was assessed with a hypergeometric test (using the “phyper” R function).

### Pathway enrichment analysis

Pathway enrichment analysis was performed for all DEGs from each dataset using MetaCore/MetaBase (GeneGo) v6.34 (Thomson Reuters, https://portal.genego.com/)^66^. The p-value for each of the 1480 human canonical pathways in MetaCore was generated using a hypergeometric test with an FDR-adj. p-value cutoff of 0.01. The Compare Experiments Workflow tool was used for comparing gene expression data across different datasets (AD/PD DEG with CMV, EBV or HHV6 DEGs) by analyzing their intersections in terms of their mappings onto MetaCore’s ontologies, including canonical pathway maps.

### Genetic variants enriched in candidate gene region

Based on the shared genes across AD or PD and CMV, EBV and HHV6 DEGs, we searched the Open Targets^80^ validation platform to identify genetic variants proximal to these candidate gene targets associated with AD or PD. Sources of genetic associations in Open Targets include the following: the GWAS catalog, Genomics England PanelApp, the PheWAS catalog, the European Variation Archive (EVA) and Gene2Phenotype^80^. Variant-gene assignment considered deleterious consequences within the gene coding region, and the variant location within introns or regulatory regions. Intergenic variants assigned to the promoter region of the nearest gene were also retrieved in this search.

### Gene expression validation in human microglia available datasets

To validate the expression profile of blood sample DEGs, publicly available datasets with human microglia gene expression datasets were identified in GEO database. Raw gene expression files were downloaded from GSE99074^40^.An initial quality check of host RNA-Seq data was performed using FastQC^81^. Quality filtered reads were mapped to the human reference genome GRCh38 ensembl 86 using STAR^82^, and quantified with featureCounts^83^. The data was annotated with Biomart^84^, and gene expression measurements were reported in log2 transcripts per million (TPM).

### Drug-target prioritization

To link putative targets (DEGs) to public compounds, we obtained evidence from approved and marketed drugs that are associated to 11,538 targets from the EMBL-EBI ChEMBL database v23^85^. This analysis included data on drugs that have been approved for marketing by the U.S. Food and Drug Administration (FDA) and direct clinical evidence of interaction with the encoded DEG.

In addition, we performed drug repurposing analysis with the Connectivity Map^45^ (CMAP, https://www.broadinstitute.org/cmap/). For each gene expression profile to host response to CMV, EBV and HHV6, the 500 genes that ranked (based on FDR-adj. p-values) at the very top and bottom of each list were selected and compared against the gene expression profiles from the Broad CMAP compound library. Significant compounds were prioritized based on anti-correlated compound enrichment score, which represents compounds inversely matched to the disease signatures surveyed (score < 0; FDR-adj. p-value ≤ 0.05; compound specificity < 0.1). To enable result interpretation, ChEMBL data^85^ on target, mechanism of action, and drug indication was integrated. Compounds with unknown mechanism of action, no clinical use or antibacterial effect were excluded from our results.

## Supplementary information

Supplementary Figures and Table Labels

Supplementary Table S1.

Supplementary Table S2.

Supplementary Table S3.

Supplementary Table S4.

Supplementary Table S5.

Supplementary Table S6.

Supplementary Table S7.

Supplementary Table S8.

Supplementary Table S9.

Supplementary Table S10.

## References

[1] Nussbaum RL, Ellis CE (2003). Alzheimer’s disease and Parkinson’s disease. N Engl J Med.

[2] Masters CL (2015). Alzheimer’s disease. Nat Rev Dis Primers.

[3] Poewe W (2017). Parkinson disease. Nat Rev Dis Primers.

[4] Zimmer R, Lauter H (1988). Diagnosis, differential diagnosis and nosologic classification of the dementia syndrome. Pharmacopsychiatry.

[5] Itzhaki RF (2017). Herpes simplex virus type 1 and Alzheimer’s disease: possible mechanisms and signposts. FASEB J.

[6] Song F, Poljak A, Smythe GA, Sachdev P (2009). Plasma biomarkers for mild cognitive impairment and Alzheimer’s disease. Brain Res Rev.

[7] Hamza TH (2010). Common genetic variation in the HLA region is associated with late-onset sporadic Parkinson’s disease. Nat Genet.

[8] van den Pol AN (2006). Viral infections in the developing and mature brain. Trends Neurosci.

[9] Deleidi M, Isacson O (2012). Viral and inflammatory triggers of neurodegenerative diseases. Sci Transl Med.

[10] Colangelo AM, Alberghina L, Papa M (2014). Astrogliosis as a therapeutic target for neurodegenerative diseases. Neurosci Lett.

[11] Henderson G, Jaber T, Carpenter D, Wechsler SL, Jones C (2009). Identification of herpes simplex virus type 1 proteins encoded within the first 1.5 kb of the latency-associated transcript. J Neurovirol.

[12] Porcellini E, Carbone I, Ianni M, Licastro F (2010). Alzheimer’s disease gene signature says: beware of brain viral infections. Immun Ageing.

[13] Westman G (2017). Decreased HHV-6 IgG in Alzheimer’s Disease. Front Neurol.

[14] Carbone I (2014). Herpes virus in Alzheimer’s disease: relation to progression of the disease. Neurobiol Aging.

[15] Hogestyn JM, Mock DJ, Mayer-Proschel M (2018). Contributions of neurotropic human herpesviruses herpes simplex virus 1 and human herpesvirus 6 to neurodegenerative disease pathology. Neural Regen Res.

[16] Wozniak M, Bell T, Denes A, Falshaw R, Itzhaki R (2015). Anti-HSV1 activity of brown algal polysaccharides and possible relevance to the treatment of Alzheimer’s disease. Int J Biol Macromol.

[17] De Chiara G (2012). Infectious agents and neurodegeneration. Mol Neurobiol.

[18] Sood S (2015). A novel multi-tissue RNA diagnostic of healthy ageing relates to cognitive health status. Genome Biol.

[19] Shamir R (2017). Analysis of blood-based gene expression in idiopathic Parkinson disease. Neurology.

[20] Riou, R. *et al*. Severe Symptomatic Primary Human Cytomegalovirus Infection despite Effective Innate and Adaptive Immune Responses. *J Virol* **91**, 10.1128/JVI.02245-16 (2017). 10.1128/JVI.02245-16 PMC5309965 28031361

[21] Nikitin PA (2010). An ATM/Chk2-mediated DNA damage-responsive signaling pathway suppresses Epstein-Barr virus transformation of primary human B cells. Cell Host Microbe.

[22] Smith N (2013). Induction of interferon-stimulated genes on the IL-4 response axis by Epstein-Barr virus infected human b cells; relevance to cellular transformation. PLoS One.

[23] Hu X, Yu J, Crosby SD, Storch GA (2013). Gene expression profiles in febrile children with defined viral and bacterial infection. Proc Natl Acad Sci USA.

[24] Hagenbuchner J (2012). FOXO3-induced reactive oxygen species are regulated by BCL2L11 (Bim) and SESN3. J Cell Sci.

[25] Johnson MR (2015). Systems genetics identifies Sestrin 3 as a regulator of a proconvulsant gene network in human epileptic hippocampus. Nat Commun.

[26] Campbell BM, Charych E, Lee AW, Moller T (2014). Kynurenines in CNS disease: regulation by inflammatory cytokines. Front Neurosci.

[27] Guillemin GJ, Brew BJ, Noonan CE, Takikawa O, Cullen KM (2005). Indoleamine 2,3 dioxygenase and quinolinic acid immunoreactivity in Alzheimer’s disease hippocampus. Neuropathol Appl Neurobiol.

[28] Widner B (2000). Tryptophan degradation and immune activation in Alzheimer’s disease. J Neural Transm (Vienna).

[29] Arner ES, Holmgren A (2000). Physiological functions of thioredoxin and thioredoxin reductase. Eur J Biochem.

[30] Arodin L (2014). Alteration of thioredoxin and glutaredoxin in the progression of Alzheimer’s disease. J Alzheimers Dis.

[31] Venkitaramani DV (2007). Beta-amyloid modulation of synaptic transmission and plasticity. J Neurosci.

[32] Arend WP, Malyak M, Guthridge CJ, Gabay C (1998). Interleukin-1 receptor antagonist: role in biology. Annu Rev Immunol.

[33] Dzwonek J, Rylski M, Kaczmarek L (2004). Matrix metalloproteinases and their endogenous inhibitors in neuronal physiology of the adult brain. FEBS Lett.

[34] Healy DG (2008). Phenotype, genotype, and worldwide genetic penetrance of LRRK2-associated Parkinson’s disease: a case-control study. Lancet Neurol.

[35] West AB (2005). Parkinson’s disease-associated mutations in leucine-rich repeat kinase 2 augment kinase activity. Proc Natl Acad Sci USA.

[36] Schapansky J, Nardozzi JD, LaVoie MJ (2015). The complex relationships between microglia, alpha-synuclein, and LRRK2 in Parkinson’s disease. Neuroscience.

[37] Kaizer EC (2007). Gene expression in peripheral blood mononuclear cells from children with diabetes. J Clin Endocrinol Metab.

[38] Pankla R (2009). Genomic transcriptional profiling identifies a candidate blood biomarker signature for the diagnosis of septicemic melioidosis. Genome Biol.

[39] Lee JM (2013). Dominant effects of the Huntington’s disease HTT CAG repeat length are captured in gene-expression data sets by a continuous analysis mathematical modeling strategy. Hum Mol Genet.

[40] Galatro TF (2017). Transcriptomic analysis of purified human cortical microglia reveals age-associated changes. Nat Neurosci.

[41] Nelson MR (2015). The support of human genetic evidence for approved drug indications. Nat Genet.

[42] Rinne JO (2002). Increased brain histamine levels in Parkinson’s disease but not in multiple system atrophy. J Neurochem.

[43] Jimenez-Jimenez FJ, Alonso-Navarro H, Garcia-Martin E, Agundez JA (2016). Thr105Ile (rs11558538) polymorphism in the histamine N-methyltransferase (HNMT) gene and risk for Parkinson disease: A PRISMA-compliant systematic review and meta-analysis. Medicine (Baltimore).

[44] Chen XX (2018). The impact of GAB2 genetic variations on cerebrospinal fluid markers in Alzheimer’s disease. Ann Transl Med.

[45] Lamb J (2006). The Connectivity Map: using gene-expression signatures to connect small molecules, genes, and disease. Science.

[46] Zandi PP, Breitner JC, Anthony JC (2002). Is pharmacological prevention of Alzheimer’s a realistic goal?. Expert Opin Pharmacother.

[47] Bettens K, Sleegers K, Van Broeckhoven C (2013). Genetic insights in Alzheimer’s disease. Lancet Neurol.

[48] Kalia LV, Lang AE (2015). Parkinson’s disease. Lancet.

[49] Lodato MA (2018). Aging and neurodegeneration are associated with increased mutations in single human neurons. Science.

[50] Perry VH, Holmes C (2014). Microglial priming in neurodegenerative disease. Nat Rev Neurol.

[51] Latta CH, Brothers HM, Wilcock DM (2015). Neuroinflammation in Alzheimer’s disease; A source of heterogeneity and target for personalized therapy. Neuroscience.

[52] Chahine LM, Stern MB, Chen-Plotkin A (2014). Blood-based biomarkers for Parkinson’s disease. Parkinsonism Relat Disord.

[53] Grunblatt E (2009). Gene expression as peripheral biomarkers for sporadic Alzheimer’s disease. J Alzheimers Dis.

[54] Liew CC, Ma J, Tang HC, Zheng R, Dempsey AA (2006). The peripheral blood transcriptome dynamically reflects system wide biology: a potential diagnostic tool. J Lab Clin Med.

[55] Maes OC (2007). Transcriptional profiling of Alzheimer blood mononuclear cells by microarray. Neurobiol Aging.

[56] Sullivan PF, Fan C, Perou CM (2006). Evaluating the comparability of gene expression in blood and brain. Am J Med Genet B Neuropsychiatr Genet.

[57] Wang R (2013). Metabolic stress modulates Alzheimer’s beta-secretase gene transcription via SIRT1-PPARgamma-PGC-1 in neurons. Cell Metab.

[58] Rosenberg GA (2009). Matrix metalloproteinases and their multiple roles in neurodegenerative diseases. Lancet Neurol.

[59] Pei Y, Banerjee S, Jha HC, Sun Z, Robertson ES (2017). An essential EBV latent antigen 3C binds Bcl6 for targeted degradation and cell proliferation. PLoS Pathog.

[60] Simon-Sanchez J (2009). Genome-wide association study reveals genetic risk underlying Parkinson’s disease. Nat Genet.

[61] Zimprich A (2004). Mutations in LRRK2 cause autosomal-dominant parkinsonism with pleomorphic pathology. Neuron.

[62] Haugarvoll K (2008). Lrrk2 R1441C parkinsonism is clinically similar to sporadic Parkinson disease. Neurology.

[63] Bae JR, Lee BD (2015). Function and dysfunction of leucine-rich repeat kinase 2 (LRRK2): Parkinson’s disease and beyond. BMB Rep.

[64] Hakimi M (2011). Parkinson’s disease-linked LRRK2 is expressed in circulating and tissue immune cells and upregulated following recognition of microbial structures. J Neural Transm (Vienna).

[65] Gardet A (2010). LRRK2 is involved in the IFN-gamma response and host response to pathogens. J Immunol.

[66] Wang Z, Arat S, Magid-Slav M, Brown JR (2018). Meta-analysis of human gene expression in response to Mycobacterium tuberculosis infection reveals potential therapeutic targets. BMC Syst Biol.

[67] Liu Z (2011). The kinase LRRK2 is a regulator of the transcription factor NFAT that modulates the severity of inflammatory bowel disease. Nat Immunol.

[68] Olson KE, Gendelman HE (2016). Immunomodulation as a neuroprotective and therapeutic strategy for Parkinson’s disease. Curr Opin Pharmacol.

[69] Xu Y (2012). Neurotransmitter receptors and cognitive dysfunction in Alzheimer’s disease and Parkinson’s disease. Prog Neurobiol.

[70] Ayutyanont N (2014). The Alzheimer’s prevention initiative composite cognitive test score: sample size estimates for the evaluation of preclinical Alzheimer’s disease treatments in presenilin 1 E280A mutation carriers. J Clin Psychiatry.

[71] Longo FM, Massa SM (2004). Neuroprotective strategies in Alzheimer’s disease. NeuroRx.

[72] Group A-FR (2015). Follow-up evaluation of cognitive function in the randomized Alzheimer’s Disease Anti-inflammatory Prevention Trial and its Follow-up Study. Alzheimers Dement.

[73] Mastroeni D (2018). Laser-captured microglia in the Alzheimer’s and Parkinson’s brain reveal unique regional expression profiles and suggest a potential role for hepatitis B in the Alzheimer’s brain. Neurobiol Aging.

[74] Lovheim H, Gilthorpe J, Adolfsson R, Nilsson LG, Elgh F (2015). Reactivated herpes simplex infection increases the risk of Alzheimer’s disease. Alzheimers Dement.

[75] Lovheim H (2015). Herpes simplex infection and the risk of Alzheimer’s disease: A nested case-control study. Alzheimers Dement.

[76] Lovheim H (2018). Interaction between Cytomegalovirus and Herpes Simplex Virus Type 1 Associated with the Risk of Alzheimer’s Disease Development. J Alzheimers Dis.

[77] Readhead B (2018). Multiscale Analysis of Independent Alzheimer’s Cohorts Finds Disruption of Molecular, Genetic, and Clinical Networks by Human Herpesvirus. Neuron.

[78] Smith SB, Magid-Slav M, Brown JR (2013). Host response to respiratory bacterial pathogens as identified by integrated analysis of human gene expression data. PLoS One.

[79] Smith SB, Dampier W, Tozeren A, Brown JR, Magid-Slav M (2012). Identification of common biological pathways and drug targets across multiple respiratory viruses based on human host gene expression analysis. PLoS One.

[80] Koscielny G (2017). Open Targets: a platform for therapeutic target identification and validation. Nucleic Acids Res.

[81] Andrews, S. FastQC: A Quality Control tool for High Throughput Sequence Data. *Available online at* https://www.bioinformatics.babraham.ac.uk/projects/fastqc/ (2010).

[82] Dobin A (2013). STAR: ultrafast universal RNA-seq aligner. Bioinformatics.

[83] Liao Y, Smyth GK, Shi W (2014). featureCounts: an efficient general purpose program for assigning sequence reads to genomic features. Bioinformatics.

[84] Durinck S, Spellman PT, Birney E, Huber W (2009). Mapping identifiers for the integration of genomic datasets with the R/Bioconductor package biomaRt. Nat Protoc.

[85] Davies M (2015). ChEMBL web services: streamlining access to drug discovery data and utilities. Nucleic Acids Res.

[86] Kumar A, Singh A (2015). & Ekavali. A review on Alzheimer’s disease pathophysiology and its management: an update. Pharmacol Rep.

[87] Blass JP, Cyrus PA, Bieber F, Gulanski B (2000). Randomized, double-blind, placebo-controlled, multicenter study to evaluate the safety and tolerability of metrifonate in patients with probable Alzheimer disease. The Metrifonate Study Group. Alzheimer Dis Assoc Disord.

[88] Heckman PR, Wouters C, Prickaerts J (2015). Phosphodiesterase inhibitors as a target for cognition enhancement in aging and Alzheimer’s disease: a translational overview. Curr Pharm Des.

[89] Lan YL, Zhao J, Li S (2015). Update on the neuroprotective effect of estrogen receptor alpha against Alzheimer’s disease. J Alzheimers Dis.

