# Supplementary material for: Shared Molecular Signatures Across Neurodegenerative Diseases and Herpes Virus Infections Highlights Potential Mechanisms for Maladaptive Innate Immune Responses
Source: Sci Rep. 2019 Jun 19;9:8795. doi: 10.1038/s41598-019-45129-8 (PMC6584587; doi:10.1038/s41598-019-45129-8)
Supplement: Supplementary file 1 — Supplementary Figures and Table Labels [file 41598_2019_45129_MOESM1_ESM.pdf]

Supplementary Note

Shared Molecular Signatures Across Neurodegenerative Diseases and Herpes Virus Infections Highlights Potential Mechanisms for Maladaptive Innate Immune Responses

Ana Caroline Costa Sa[1], Heather Madsen[2], James R. Brown[1*]

[1] Computational Biology, Human Genetics, Research and Development (R&D), GlaxoSmithKline (GSK), Collegeville, PA 19426, USA
[2] HIV Discovery, ViiV Healthcare, Research Triangle Park, NC 27713, USA

* Correspondence to:
James R. Brown (James.R.Brown@gsk.com)
1250 S. Collegeville Road, Collegeville, Pennsylvania, 19426-0989, United States
Tel: +1 (610) 247-8580

Supplementary Figures and Table Labels

Supplementary Figure S1. Control analyses for disease relevance of detected molecular signatures.

Supplementary Table S1. Gene expression profile datasets reviewed and the criteria for their inclusion or exclusion from analysis.

Supplementary Table S2. Significant differentially expressed genes in relation to Alzheimer's Disease and CMV human host response.

Supplementary Table S3. Significant differentially expressed genes (FDR adjusted $P<0.05$) in relation to Alzheimer's Disease and EBV human host response.

Supplementary Table S4. Significant differentially expressed genes (FDR adjusted $P<0.05$) in relation to Alzheimer's Disease and HHV6 human host response.

Supplementary Table S5. Human canonical pathways significantly enriched from DEGs associated with Alzheimer's Disease and human host response to CMV, EBV and HHV6.

Supplementary Table S6. Significant differentially expressed genes (FDR adjusted $P<0.05$) in relation to Parkinson's Disease and CMV human host response.

Supplementary Table S7. Significant differentially expressed genes (FDR adjusted $P<0.05$) in relation to Parkinson's Disease and EBV human host response.

Supplementary Table S8. Significant differentially expressed genes (FDR adjusted $P<0.05$) in relation to Parkinson's Disease and HHV6 human host response.

Supplementary Table S9. Human canonical pathways significantly enriched from DEGs associated with Parkinson's Disease and human host response to CMV, EBV and HHV6.

Supplementary Table S10. List of 20 DEGs associated with viral host response and Alzheimer’s Disease or Parkinson’s Disease proximal to SNPs associated with type 2 Diabetes in the GWAS catalog.

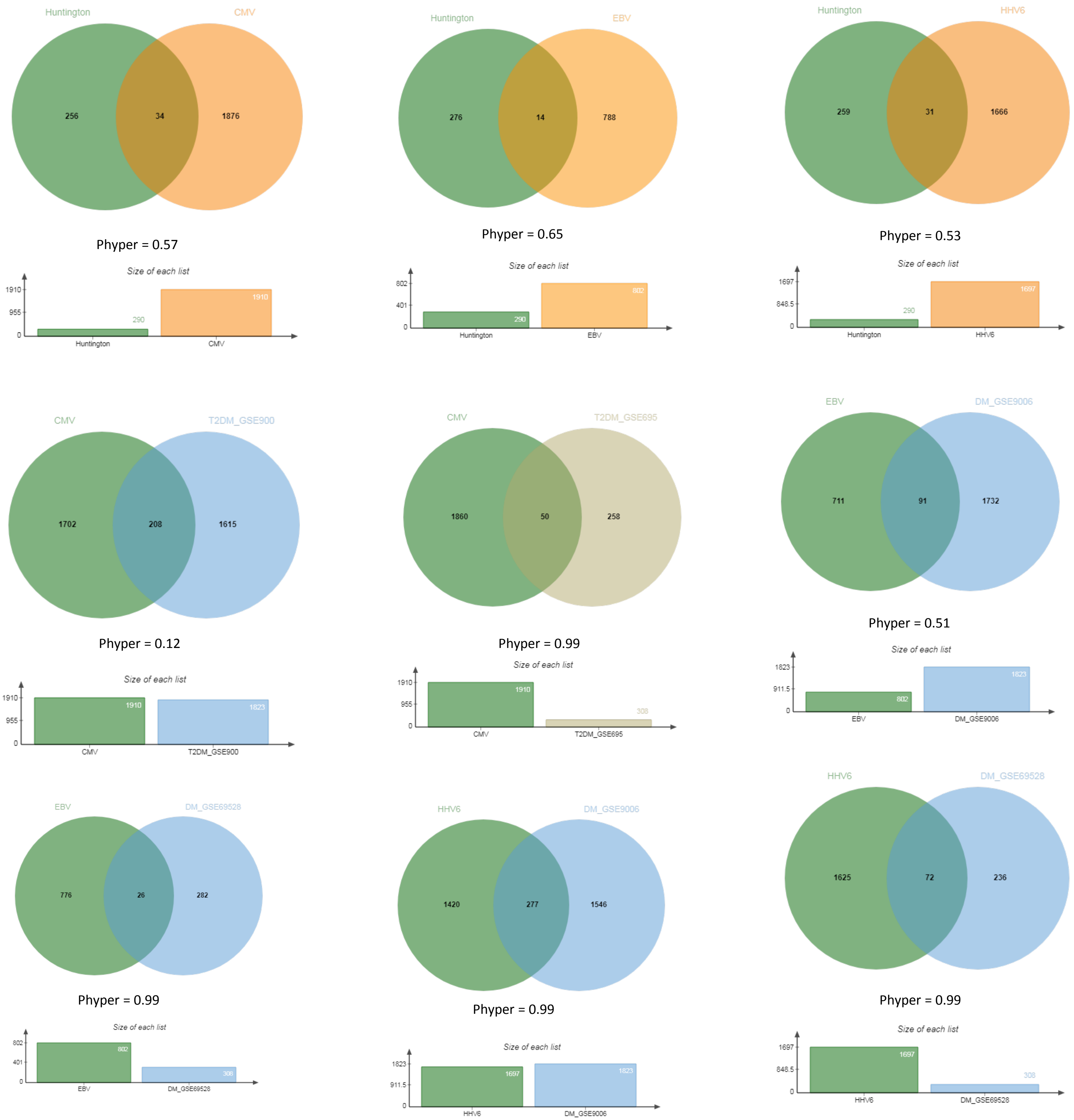

Supplementary Fig 1. Control analyses for disease relevance of detected molecular signatures. Comparisons of CMV, EBV and HHV6 host response DEGs to gene associated with three different human gene expression datasets for Huntington's Disease (HD) and for Type 2 diabetes mellitus (T2DM; FDR adj. P<0.05; See Materials and Methods). No statistically significant enrichment occurred for HD or T2DM (Phyper > 0.1) which supports that common molecular signatures between AD or PD and Herpesviridae infections are non-spurious
